# Parental stress around ophthalmological health conditions: a systematic review of literature protocol

**DOI:** 10.1186/s13643-021-01773-8

**Published:** 2021-08-13

**Authors:** Julio Cesar Souza-Silva, Cleusa Alves Martins, Marco Túlio Antônio Garciazapata, Maria Alves Barbosa

**Affiliations:** 1 Faculty of Medicine, Post-Graduate Program in Health Science, Federal University of Goiás, Secretaria–1a s/n–Setor Universitário, Goiânia, Goiás 74605-020 Brazil; 2 Faculty of Nursing, Federal University of Goiás, Rua 227 qd. 68 Setor Leste Universitário, Setor Leste Universitário 74605080, Goiânia, GO Brazil; 3 Department of Tropical Medicine and Dermatology, Institute of Tropical Pathology and Public Health, Rua 235/Esq. com 1a Avenida, Setor Universitário 74605050, Caixa-postal: 131, Goiânia, GO Brazil

**Keywords:** Parenting Stress, Ophthalmology, Ocular Diseases, Systematic Review, Eye Diseases

## Abstract

**Background:**

Parents can be psychologically impacted when their children are diagnosed with eye diseases, such as blindness, strabismus, and eye cancer. Stress can reduce the quality of parental care and may be linked to the deterioration of parents’ and children’s mental and physical health and family dynamics. No systematic literature review on parental stress in ophthalmology has been found to provide evidence synthesis capable of stimulating and defining new studies and thereby promoting research in this field. To address this important gap, the present review aims to synthesize evidence about approaches, methods, instruments, and results from research regarding ophthalmology-related parental stress.

**Methods:**

Primary epidemiological observational studies should be original in addressing parental stress caused by ophthalmological health conditions in children. They should present the characteristics of the study population and the clinical and ophthalmic characterizations of children.

MEDLINE (via Ovid), EMBASE, PsycINFO, Google Scholar, and gray literature (PsycEXTRA, NTIS, and OpenSINGLE) will be searched. Controlled vocabulary, Boolean operators, and defined search strategies will be used. There will be no restrictions on the studies’ publication language, which will be selected in two screening stages. Two reviewers will independently retrieve full-text studies, assess methodological quality, and extract data. Data available through December 2021 will be considered for inclusion.

**Discussion:**

The socioeconomic characterization of the participants, the identification of which ophthalmological diseases have been studied in relation to parental stress, and the knowledge of each instrument and methodology peculiarities potentially contribute to this study. The results may promote the development or enhancement of public policies focused on this specific theme, thereby providing the means for potential improvement of the physical and mental health of parents and children with eye diseases.

Systematic review registration

PROSPERO CRD42018094972

**Supplementary Information:**

The online version contains supplementary material available at 10.1186/s13643-021-01773-8.

## Background

### Description of the disease

The World Health Organization estimates that 1.4 million children—defined as individuals between 0 and 15 years of age—are blind, with a prevalence of 0.3/1000 children in developed countries and 1.5/1000 in poor/very poor communities. These children face a lifetime of blindness, which equates to a combined estimated 75 million blind years (blind individuals × life expectancy) [1].

Each year, 500,000 children (approximately one per minute) are born blind or become blind before their fifth birthday. The causes of blindness, which vary according to geographic region and socioeconomic status, include corneal scarring, cataracts, glaucoma, retinopathy of prematurity, and refractive errors. Although eye disease may not always lead to low vision or blindness, it can still cause significant parental stress stemming from the processes of diagnosis, treatment, and rehabilitation of vision. In all such cases, parental involvement and understanding are of paramount importance [1].

Being the parent of a child with any chronic disease can cause stress [2, 3], which is defined by Hans Selye as “a non-specific body response to any demand made upon it” [4]. Parental stress refers to a series of processes that lead to psychological and physiological reactions when attempting to adapt to parenting activities [3]. Early identification of increased stress in the parent–child system and suitable interventions can help reduce stress and diminish the frequency and intensity of the child’s emotional and behavioral disorders [5]. The reduction of parental stress improves parental health [6] and positively impacts the quality of care provided to sick children [7].

Conversely, increasing and chronic levels of parental stress may put parents, children, and other family members at risk of adverse physical and psychological effects, such as anxiety and depression [8]. Among children, prolonged stress can lead to social incompetence, maladaptive behaviors, and cognitive impairment [2]. Stressful events often influence the pathogenesis of physical illness, causing negative affective states. In turn, these directly impact biological processes or care patterns, increasing the risk of becoming sick [9].

Changes in care may occur due to caregivers’ adaptations or coping responses to stress, such as increased smoking, decreased physical activity, insomnia, disinterest, and poor adherence to medical prescriptions and healthy habits. Furthermore, stress has been linked to major depression and poor adherence to treatments [9].

There are records of interference in the parent–child system due to parental stress around a child’s ophthalmological issues, but we did not find any systemic literature reviews on the subject. The current published studies on psychological/psychiatric diseases in the ophthalmological field [10–17] mainly focus on depression, anxiety, and parental burden; they do not address parental stress and child with visual disorders. A systematic literature review aimed at generating evidence and synthesis of what has been studied and published is required to support the conception and planning of new studies. The evidence synthesis will guide new research and promote further studies to improve care for children with eye diseases along with the physical and mental health of their parents.

### Eye disorders in children and psychological implications for parents and family

During pregnancy, parents idealize the birth of a perfect child [18]. The birth of a blind child with strabismus, glaucoma, or congenital eye diseases can create a discrepancy between the idealized and the real child. One aspect of motherhood necessary to establish a healthy mother–child relationship is being able to deal with such discrepancies. Failure in overcoming expectation–reality discrepancies can lead mothers to become depressed, distance themselves from the child, and become unable to provide the warmth and love required to promote the child’s healthy development [10].

The establishment of an official diagnosis of a child with disability (e.g., blindness) marks the occurrence of a family crisis, where members start expressing feelings of sadness, anger, guilt, helplessness, and isolation. Stress can be linked to the disruption of what was idealized versus the reality and the breakdown of family routine, and parents with very high levels of stress are required to be guided to enable them to effectively provide higher levels of care [19].

The possibility of parental stress becoming an agent of change in the relationship between a parent and a child raises questions as to what levels of resilience and coping strategies must be adopted by children’s families to alleviate the possible psychological distress caused by children’s eye diseases. It is unclear whether stress associated with the basal parental role of daily life is sufficiently robust to lead to clinical disturbances or whether the parental stress experienced by the parents of children with eye diseases correlates with psychological conditions that are potentially harmful to the parents, child, or family health.

More research about parental stress related to ophthalmological disorders in children is necessary because (1) blindness affects over a million children worldwide, (2) stress is a known contributor to many serious health issues, and (3) quality of life and appropriate allocation of healthcare resources are high-priority issues. Systematic reviews will help translate knowledge into action and promote more relevant studies. The first step to progress research in this field is to create a protocol for the synthesized evidence on the methodology used by researchers to evaluate parental stress related to children’s eye diseases, the research instruments, the psychometric characteristics of these conditions, and study design advantages and disadvantages, limitations, and peculiarities.

### Review questions

The review questions for this systematic review of literature are as follows:

i. What approaches have been used to research parental stress resulting from pediatric eye disease?
ii. What methodologies are employed in studies of parental stress resulting from pediatric eye diseases?
iii. What parental stress assessment tools are used in pediatric eye disease research?
iv. What peculiarities and psychometric characteristics of parental stress assessment instruments are employed in pediatric eye disease research?
v. What are the main results of research conducted on parental stress resulting from pediatric eye diseases?

### Objectives

- To identify studies and their respective authors as well as the bibliographical references related to parental stress in ophthalmology
- To characterize the sociodemographic aspects of participants in studies on parental stress in ophthalmology as well as the clinical and ophthalmic conditions of their children
- To identify the methodological design and timeline of selected studies on parental stress in ophthalmology
- To describe the methodology of the instrument—if present—used to measure parental stress, with emphasis on its psychometric characteristics (internal reliability, test and retest reliability, and validation criteria)
- To highlight the main results and conclusions of authors of published research on parental stress around ophthalmology
- To detect the peculiarities of each study presented in the research on parental stress around ophthalmology

### Inclusion criteria

Population, exposure, comparator, outcome, and study design components (PECOS) to be analyzed in the studies are as follows:

i. *P*opulation/participants: father, mother, or both
ii. *E*xposure of interest: eye disease in the child resulting from severe reduction in visual acuity, dysfunction of extrinsic eye motility, cancer, congenital eye diseases, deforming/stigmatizing eye, and periocular changes or any eye disease with the potential to increase parental stress
iii. *C*omparator: no specific comparator
iv. *O*utcome: parental stress
v. *S*tudy design: primary epidemiological observational studies

Studies should be original in addressing parental stress (only father, mother, or both parents) caused by eye disease in children. The terms “parental stress” or “parenting stress” or similar terms describing this condition must be included in the title, summary, or the full text. Characterization of the study population and clinical and ophthalmic characterization of the children should be present. Regarding the type of research, laboratory experimental studies, reviews, editorials, comments, mathematical models, methodological articles, expert opinions, and other methodological modalities will be excluded due to the methodological differences that may hinder comparison across studies. Studies on parental stress in caregivers or other family members will also not be eligible. Similar studies that do not meet all the inclusion criteria, but that have relevant information, will be analyzed separately in the discussion section of the systematic review.

## Methods

The proposed literature review will be conducted in accordance with the Joanna Briggs Institute methodology for systematic reviews of observational epidemiological studies that report prevalence and cumulative incidence data [20].

### Study record

This systematic literature review protocol is an integral part of a doctoral dissertation, entitled “Parental Stress in Mothers and Quality of Life of Infants and Blind Children.” It has been registered in the international database PROSPERO (under registration number CRD42018094972) and is being reported according to the *Preferred Reporting Items for Systematic Reviews and Meta-Analyses Protocols* (PRISMA-P) [21, 22] (see checklist in Additional File 1 ).

### Information sources

The research strategy will be developed using *Medical Subject* *Headings* (MeSH) and *Elsevier Life Science Thesaurus (Emtree)* descriptors. Reviewers (JCSS and CAM) will systematically search the following databases: MEDLINE (via Ovid), EMBASE, PsycINFO, and Google Scholar.

Other data sources, such as ProQuest Dissertations and Theses, databases, and gray literature (i.e., PsycEXTRA, NTIS and OpenSINGLE), will also be consulted. Data will be considered for inclusion until December 2021.

### Search strategy

Searches will use a controlled vocabulary defined by initial search terms. Examples of search terms and search strategies are presented in Additional File 2. The snowballing method [23] will be used to identify other studies from the references of selected articles. Search terms in Emtree will be matched with equivalent terms in MEDLINE, to be able to conduct a search in EMBASE. Searches will be designed to be conducted in MEDLINE and EMBASE and will be adapted to other electronic databases and gray literature, always aiming to achieve search equivalence. There is no time limit filter for the inclusion of articles in all databases. The search strategy will be developed by an information science expert, aiming at conducting a high specificity and sensitivity search for the review.

### Study selection

Studies should be original in addressing parental stress (only father, mother, or both parents) caused by eye disease in children. Parental stress (or parenting stress) must be written in the title, summary, or the full text. Additionally, the characterization of the study population and clinical and ophthalmic characterization of the children should be present. The study search will be conducted by two independent researchers [24] in a two-stage selection process.

There will be no blind strategy for reviewers regarding authors’ names, institutional affiliations, or country of origin of the analyzed studies.

The raw data obtained from the databases will be processed and duplicate studies eliminated using the ENDNOTE® (ENDNOTE X7, Thomson Reuters, USA) reference manager program.

#### Selection phase I

In the first phase, the articles will undergo Relevance Test 1 (RT-1); reviewers will analyze the title and abstract of the article, determining whether it fulfills the eligibility criteria listed in Table 1.

**Table 1:** Relevance Test 1 (RT-1) of studies included in the systematic review

	**Yes**	**Not clear**	**No**
Is the research original?			
Does the population include only fathers, only mothers, or both parents?			
Does the child have an eye disease/condition?			
Does the child’s condition result in parental stress?			
Is the child between 0 and 12 years old?			
Does the child have another chronic/disabling condition?			

#### Selection phase II

After meeting the RT-1 criteria, studies will be subjected to Relevance Test 2 (RT-2). This verifies, more strictly, if there is presence of full text, characterization of the study population, and clinical and ophthalmic characterization of the children (see RT-2 in Table 2).

**Table 2:** Relevance test 2 (RT-2) for the systematic literature review

	**Yes**	**Not clear**	**No**
Is there an instrument that specifically assesses parental stress?			
Is the full text of the article available in databases?			
Is there a characterization of the study population?			
Is there a methodological characterization of the instrument?			
Is there a clinical and ophthalmic characterization of the children?			

Excluded studies will be recorded in a separate table along with the reason for exclusion. “Results” will include a comment section containing the characteristics of each excluded study and the reason that led to their exclusion.

In phases I and II, articles having items evaluated as “not clear” will be the objects of a joint analysis among reviewers. In cases where disagreement persists, a third reviewer will provide his/her opinion essential to the resolution of the case. Studies that meet all the inclusion criteria will be accepted for research. The agreement achieved among the reviewers of this systematic review will be assessed using Cohen’s (*k*) kappa agreement index [25].

**Fig. 1:**
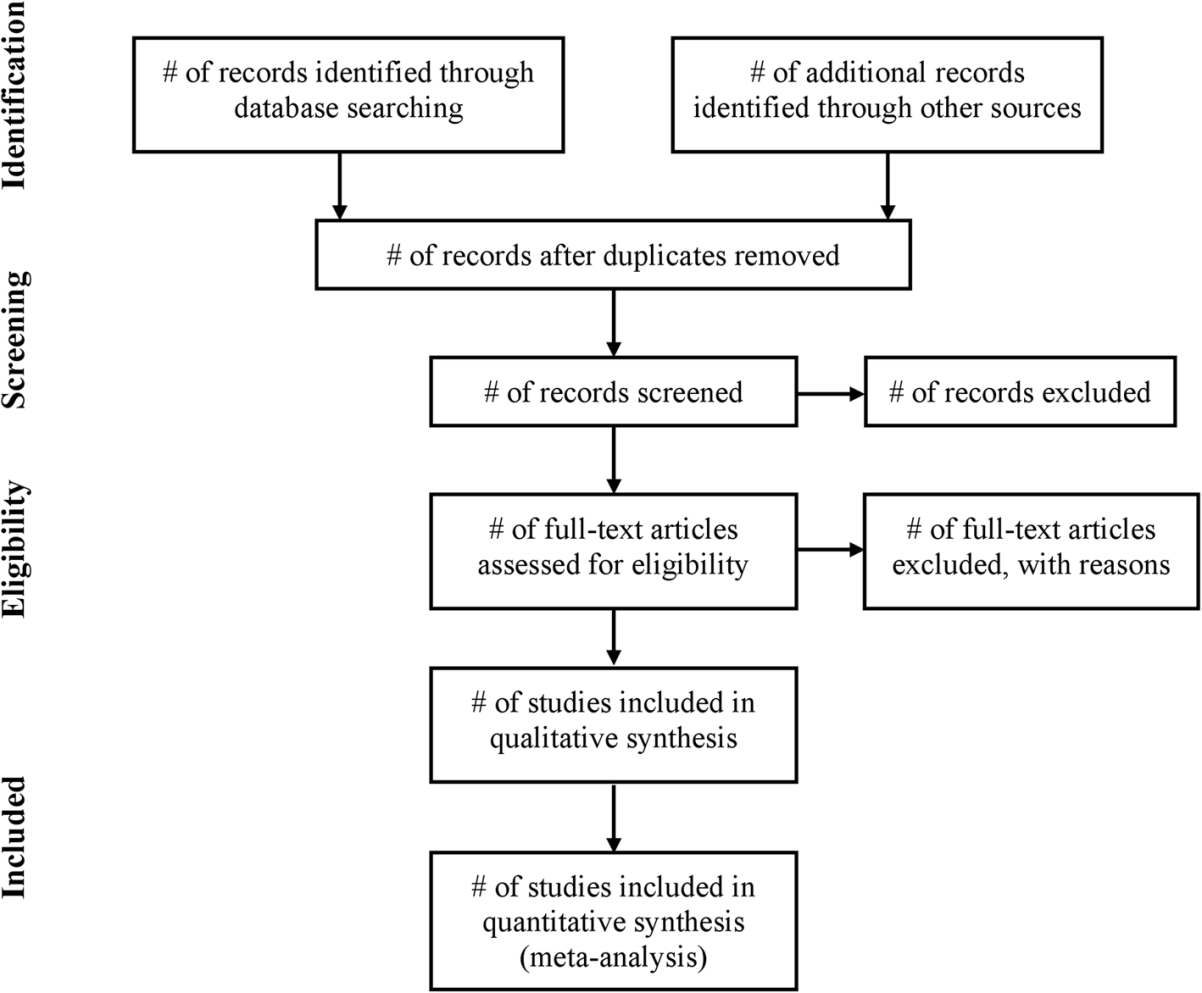
Preferred Reporting Items for Systematic Reviews and Meta-analyses (PRISMA) flow diagram

The results of the search will be reported in full in the final report and presented in a Preferred Reporting Items for Systematic Reviews and Meta-analyses (PRISMA) flow diagram (see Fig. 1) [26].

### Assessment of methodological quality

The quality of the included articles will be assessed using the Joanna Briggs Institute assessment tool for methodological guidance for systematic reviews of observational epidemiological studies reporting prevalence and cumulative incidence data (see Additional File 3) [20].

The methodological quality of the included articles will be assessed by two reviewers. In case of disagreement regarding the quality of a particular study, a third reviewer will be the tiebreaker, providing the final assessment. There is no intention of conducting a meta-analysis of the data.

### Data extraction and management

#### Data extraction

Data will be extracted from papers included in the systematic review by the two independent reviewers using a data extraction tool developed by the reviewers. In this phase, the text of the article will be analyzed in full, elaborating a standardized cataloging form to record product information (see form in Additional File 4 ).

Additional relevant information not foreseen in the fields above and the peculiarities of each study will be recorded in the “observations” field.

If some information or detail is missing from the article, the study will be included, although the absence of data will be noted.

In case of lack/loss/uncertainty of information, the author of the study will be contacted by email three times. If the problem persists, the study will be excluded.

#### Data synthesis

Since our study focuses on methodological analysis by assessing approaches, methodologies, instrument characteristics, and psychometric characteristics, there will be no combination of individual outcomes—that is, no statistical calculations or meta-analysis of results will be performed. Heterogeneity will be verified through the analysis of subgroups and configurations (country, hospital, clinic, etc.), conditions (complete blindness, sudden onset versus gradual loss of vision), population (children, preschool age, adolescents), observing possible changes in the approach or in the type of instrument to be administered to measure stress. This will generate a measurement capable of summarizing the outcomes. We will critically analyze the data and quality of the studies included, providing descriptive tabulations and summaries. The results extracted from the cataloging form will be categorized and compared between the different studies. If any included studies show important methodological differences, a sensitivity analysis will be carried out. Gaps and limitations in the description or methodology will be noted and discussed.

### Protocol changes

Any substantive change to this protocol will be recorded upon its occurrence, in PROSPERO, and documented in the final publication.

## Discussion

To our knowledge, this will be the first systematic literature review related to the comparative assessment of approaches, methods, and results in the research on parental stress around ophthalmology. Systematic analysis and synthesis of peculiarities, time of administration, cost, reliability, and stability can offer researchers support for instrument analysis for choosing the best match for their research. Psycho-ophthalmology is a field linking ophthalmology and psychiatry [27]. Few studies related to parental stress have been conducted on this subject. The socioeconomic characterization of the participants, the identification of which ophthalmological diseases have been studied in relation to parental stress, and the knowledge of each instrument and methodology peculiarities potentially contribute to this study. The results may promote the development or enhancement of public policies focused on this specific theme, thereby providing the means for the potential improvement of the physical and mental health of parents of and children with eye diseases.

This review protocol may have potential limitations if attempted to predict the evaluation of psychometric characteristics, such as equivalence and construct analysis [28]; however, as it does not attempt to do so, it keeps the specific evaluation as the object of study of future research.

## Supplementary Information

**Additional file 1.** PRISMA-P Checklist. This file contains the completed PRISMA-P checklist.

**Additional file 2.** Search strategy and search terms for MEDLINE (Ovid). This file presents the search strategy and terms to be used in the review.

**Additional file 3.** Joanna Briggs Institute assessment tool for methodological guidance for systematic reviews of observational epidemiological studies reporting prevalence and cumulative incidence data. This file presents the Joanna Briggs Assessment of Methodological Quality Tool.

**Additional file 4.** Standardized form for data collection. This file presents the form for data collection.

## Data Availability

Not applicable.

## Abbreviations

CAPES: *Coordenação** de **Aperfeiçoamento** de **Pessoal** de **Nível** Superior* (Coordination for the Improvement of Higher Education Personnel)
FAPEG: *Fundação** de **Amparo** à **Pesquisa** do Estado de **Goiás** (*Research Support Foundation of the State of Goiás)
