# Supplementary material for: Parental stress around ophthalmological health conditions: a systematic review of literature protocol
Source: Syst Rev. 2021 Aug 13;10:228. doi: 10.1186/s13643-021-01773-8 (PMC8364077; doi:10.1186/s13643-021-01773-8)
Supplement: Supplementary file 2 — Additional file 2. Search strategy and search terms for MEDLINE (Ovid). This file presents the search strategy and terms to be used in the review. [file 13643_2021_1773_MOESM2_ESM.docx]

Search strategy and search terms for MEDLINE (Ovid)

**1. Search Strategy for MEDLINE (Ovid) : Pilot test conducted on December 25, 2019**

| # | Searchs | Results |
| --- | --- | --- |
| 1 | (Vis* Problem$ or Vis* Impair*).ti,ab. or Vision Disorders/ or visual impairment/ | 40,393 |
| 2 | ((Parent* Stress or Parent* distress or Caregiv* Stress or Caregiv* distress).ti,ab. or (*intervention/ and *parent-child relationship/ )) | 6,013 |
| 3 | ((mother$ OR father$ or child* or infan* or cataract$ or aphak* or surgery or glaucom*).ti,ab. or *VIPP/) | 3,117,430 |
| 4 | 1 and 2 and 3 | 15 |
| 5 | (Caregiver$ and Cataract$ and parent*).ti,ab. | 20 |
| 6 | (Humans/ and Male/ and Female/ and Infant/ and Stress, Psychological/) | 2,089 |
| 7 | 5 and 6 | 3 |
| 8 | (infant$ and Cataract$ and aphak*).ti,ab | 295 |
| 9 | (Humans/ and Male/ and Female/ and Infant/ and aphakia/) | 39 |
| 10 | 8 and 9 | 7 |
| 11 | (Cataract/cn AND Cataract Extraction AND Aphakia, Postcataract/th) | 71 |
| 12 | (Research Design AND Cataract Extraction AND Follow-Up Studies AND Treatment Outcome).mp. | 2 |
| 13 | 11 and 12 | 1 |
| 14 | Caregivers/px and Cataract/cn and Cataract/th and Parents/px and Stress, Psychological and Surveys and Questionnaires | 1 |
| 15 | Anxiety/px and Blindness/px and Child and Disabled Children and Parents/px | 1 |
| 16 | 4 or 7 or 10 or 13 or 14 or 15 | 25 |
| 17 | remove duplicates from 16 | 21 |
| 18 | 22491410.ui. or 29359632.ui or 30148877.ui | 3 |
| 19 | 17 and 18 | 24 |

**2. Search Terms**

| SEARCH TERMS ACCORDING TO PECOS | |
| --- | --- |
| Participants | *father, mother*, OR *parent* |
| Exposure^,1^ | *child, children, childhood,* OR *congenital*, AND *blind, blindness, glaucoma, glaucomatous, squint, cataract, low vision, vision loss, retinitis pigmentosa, retinopathy of prematurity, refractive error, corneal scar, amblyopia, anophthalmia, hypertelorism, orbit dystopia, eye malformation, eye disease*, OR *crooked eye* |
| Comparator | *methodology,* *psychological tests* [MeSH], *surveys and questionnaires* [MeSH], *assessment, instrument, scale, psychometrics* [MeSH], *psychometry, reliability, validity, results,* OR *characteristic* |
| Outcomes | *stress, psychological* [MeSH], *stress,* OR *distress* AND *parenting* OR *parental* |
| Study design | *observational, cohort, retrospective, cross-sectional,* AND *nonrandomized.* |

^1^ Among these, there are some major causes of childhood blindness according to the World Health Organization and terms related to potentially stigmatizing diseases for children.
