# Supplementary material for: Parental stress around ophthalmological health conditions: a systematic review of literature protocol
Source: Syst Rev. 2021 Aug 13;10:228. doi: 10.1186/s13643-021-01773-8 (PMC8364077; doi:10.1186/s13643-021-01773-8)
Supplement: Supplementary file 3 — Additional file 3. Joanna Briggs Institute assessment tool for methodological guidance for systematic reviews of observational epidemiological studies reporting prevalence and cumulative incidence data. This file presents the Joanna Briggs Assessment of Methodological Quality Tool. [file 13643_2021_1773_MOESM3_ESM.docx]

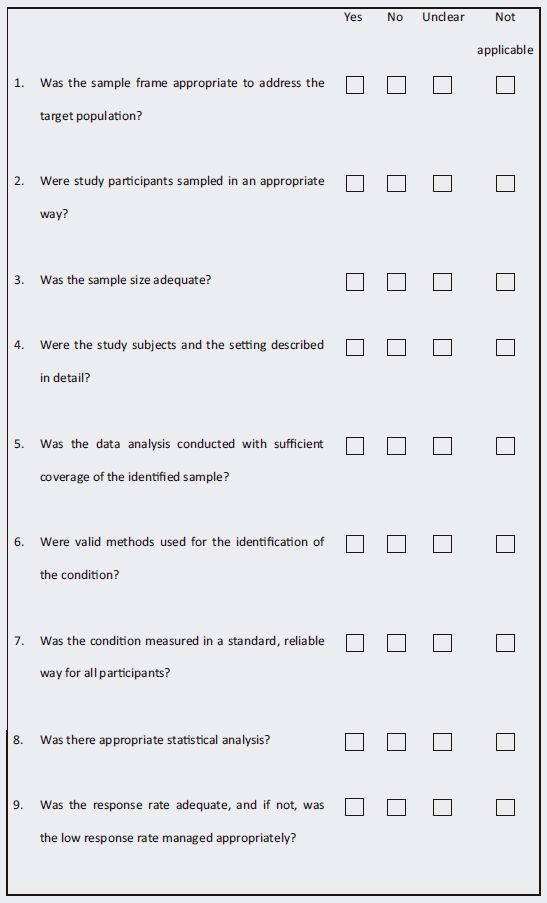
Joanna Briggs Institute assessment tool for methodological guidance for systematic reviews of observational epidemiological studies reporting prevalence and cumulative incidence data
