# Supplementary material for: Parental stress around ophthalmological health conditions: a systematic review of literature protocol
Source: Syst Rev. 2021 Aug 13;10:228. doi: 10.1186/s13643-021-01773-8 (PMC8364077; doi:10.1186/s13643-021-01773-8)
Supplement: Supplementary file 4 — Additional file 4. Standardized form for data collection. This file presents the form for data collection. [file 13643_2021_1773_MOESM4_ESM.docx]

| Author: |
| --- |
| Title of Article: |
| Review: |
| Year: |
| Reference: |
| Name of the instrument used to assess parental stress: |
| Acronym for the instrument used to assess parental stress: |
| Language(s) in which the instrument has been translated and validated: |
| Characteristics of the study population: |
| Clinical and ophthalmic characteristics: |
| Methodological characteristics of the study: |
| Characteristics of the instrument used to assess parental stress:   - Number of questions: - Time of administration: - Type of questions: - Number of domains: - Cronbach’s α: - Test-retest reliability: - Retest time: |
| Instrument methodology: |
| Validation criteria: |
| Parental stress specificity: |
| Characteristics of the study population: |
| Main results of the study: |
| Main conclusions of the study: |
| Observations: |

Standardized form for data collection
